# Effects of Signal Peptide and Chaperone Co-Expression on Heterologous Protein Production in *Escherichia coli*

**DOI:** 10.3390/molecules28145594

**Published:** 2023-07-23

**Authors:** Juntratip Jomrit, Suhardi Suhardi, Pijug Summpunn

**Affiliations:** 1 School of Pharmacy, Walailak University, Nakhon Si Thammarat 80160, Thailand; 2 Department of Animal Science, Faculty of Agriculture, Mulawarman University, Samarinda 75123, Indonesia; 3 Food Technology and Innovation Research Center of Excellence, School of Agricultural Technology and Food industry, Walailak University, Nakhon Si Thammarat 80160, Thailand

**Keywords:** signal peptides, chaperone, heterologous proteins, productivity

## Abstract

Various host systems have been employed to increase the yield of recombinant proteins. However, some recombinant proteins were successfully produced at high yields but with no functional activities. To achieve both high protein yield and high activities, molecular biological strategies have been continuously developed. This work describes the effect of signal peptide (SP) and co-expression of molecular chaperones on the production of active recombinant protein in *Escherichia coli*. Extracellular enzymes from *Bacillus subtilis*, including β-1,4-xylanase, β-1,4-glucanase, and β-mannanase constructed with and without their signal peptides and intracellular enzymes from *Pseudomonas stutzeri* ST201, including benzoylformate decarboxylase (BFDC), benzaldehyde dehydrogenase (BADH), and d-phenylglycine aminotransferase (d-PhgAT) were cloned and overexpressed in *E. coli* BL21(DE3). Co-expression of molecular chaperones with all enzymes studied was also investigated. Yields of β-1,4-xylanase (Xyn), β-1,4-glucanase (Cel), and β-mannanase (Man), when constructed without their N-terminal signal peptides, increased 1112.61-, 1.75-, and 1.12-fold, respectively, compared to those of spXyn, spCel, and spMan, when constructed with their signal peptides. For the natural intracellular enzymes, the chaperones, GroEL-GroES complex, increased yields of active BFDC, BADH, and d-PhgAT, up to 1.31-, 4.94- and 37.93-fold, respectively, and also increased yields of Man and Xyn up to 1.53- and 3.46-fold, respectively, while other chaperones including DnaK-DnaJ-GrpE and Trigger factor (Tf) showed variable effects with these enzymes. This study successfully cloned and overexpressed extracellular and intracellular enzymes in *E. coli* BL21(DE3). When the signal peptide regions of the secretory enzymes were removed, yields of active enzymes were higher than those with intact signal peptides. In addition, a higher yield of active enzymes was obtained, in general, when these enzymes were co-expressed with appropriate chaperones. Therefore, *E. coli* can produce cytoplasmic and secretory enzymes effectively if only the enzyme coding sequence without its signal peptide is used and appropriate chaperones are co-expressed to assist in correct folding.

## 1. Introduction

To produce recombinant proteins, various types of hosts, including eukaryotes such as plants, mammalian cells, insect cells, filamentous fungi, and yeast, or prokaryotes such as *Escherichia coli* and *Bacillus* sp. are employed [1,2,3]. To decide which production system is most suitable, the nature, origin, and final application of the target proteins must be considered. For example, if a downstream process modification such as glycosylation is not essential for bioactivity, bacterial expression systems are attractive for heterologous protein production because of their ability to grow rapidly and to reach high cell density using inexpensive substrates, as well as their genetics are well-characterized. Furthermore, a large number of cloning vectors and modified host strains are available [3]. In this study, we described the use of molecular biological strategies, including the alternation of secretory proteins to allow proper cytoplasmic folding and co-expression of molecular chaperones to assist the folding in order to improve the production of active recombinant proteins in the bacterial host, *E. coli*. It was reported that DnaK-DnaJ-GrpE or Trigger factor (Tf) assisted in increasing the solubility of some proteins at the early stages of the protein folding pathway [4], while GroEL-GroES was required at a later folding stage [5]. The chaperones were also reported to minimize protein aggregation by mediating the degradation of proteins that cannot be properly folded, as observed with some proteins when co-expressed with chaperones [6]. The recombinant proteins of interest in this study were enzymes involved in non-starch polysaccharide hydrolases; β-1,4-xylanase (Xyn; EC 3.2.1.8), β-1,4-glucanase (Cel; EC 3.2.1.4) and β-mannanase (Man; EC 3.2.1.78) and those involved in aromatic compound degradation pathway; benzoylformate decarboxylase (BFDC; EC 4.1.1.7) and benzaldehyde dehydrogenase (BADH; EC 1.2.1.28), as well as that involved in a reversible transamination reaction; d-phenylglycine aminotransferase (d-PhgAT; EC 2.6.1.72).

The non-starch polysaccharide hydrolases are those used in industrial processes such as bio-bleaching of pulp wood in the paper industry [7], reduction of gum content in the textile industry [8], preparation of animal feed as animal feed additives, preparation of certain compounds for the cosmetic and pharmaceutical industries [9], extraction of oils from leguminous seeds, reduction of viscosity in coffee manufacture [10], clarification of fruit juices, fabrication of sugar beet syrup and caramel and fruit flavors for numerous food industries [9,11,12]. The enzymes involved in the aromatic compound degradation pathway, BFDC, and BADH, were found to play roles in the catabolic pathways of mandelate [13,14] and d-phenylglycine [15]. They have been used for the synthesis of enantiomerically pure pharmaceutical and chemical compounds [14,16], which are important pharmaceutical agents for the production of, for example, anti-cancer drugs and analgesics such as aspirin and cocaine [17]. Lastly, d-PhgAT was reported to play a role in d-phenylglycine degradation pathway [15]. The enzyme has also been used for the synthesis of d-phenylglycine (d-Phg) or d-4-hydroxyphenylglycine (d-4-OHPhg) using L-glutamate as an amino-group donor, and itself is converted 2-oxoglutarate. d-Phg is the side chain of semisynthetic penicillins and cephalosporins such as ampicillin, cephalexin, cephaloglycine, and cefacro; whereas, d-4-OHPhg is the side-chain of amoxicillin, cephadroxil, and cefatrizine. Furthermore, d-PhgAT has been used to measure L-glutamate in food products [18], and recently, it has also been used to determine the vitamin B6 status by measuring pyridoxal-5′-phosphate in plasma samples [19].

Upon overexpression of heterologous proteins in *E. coli*, inclusion body formation and proteolytic degradation are commonly observed due to the differences in the cellular environment, folding machinery, and conformational quality control checkpoints of *E. coli* compared to those of the native hosts. To alleviate these problems, a number of approaches, including (1) reducing the rate of the target gene expression by using weaker promoters, (2) decreasing the concentration of inducer, (3) lowering the growth temperature so that transcription and translation rates are slowed down, and the strength of hydrophobic interactions that contribute to protein misfolding was reduced and (4) The use of plasmid with low copy number has been considered to attenuate the expression. However, these strategies cause a reduction in productivity. Newer approaches are the use of a hydrophilic affinity tag to decrease the inclusion body formation and the co-expression of molecular chaperones. In this study, co-expression of molecular chaperones was used to improve the yields of soluble proteins in the *E. coli* cytoplasm.

## 2. Results and Discussion

### 2.1. Construction of Recombinant Plasmids

**Figure 1:**
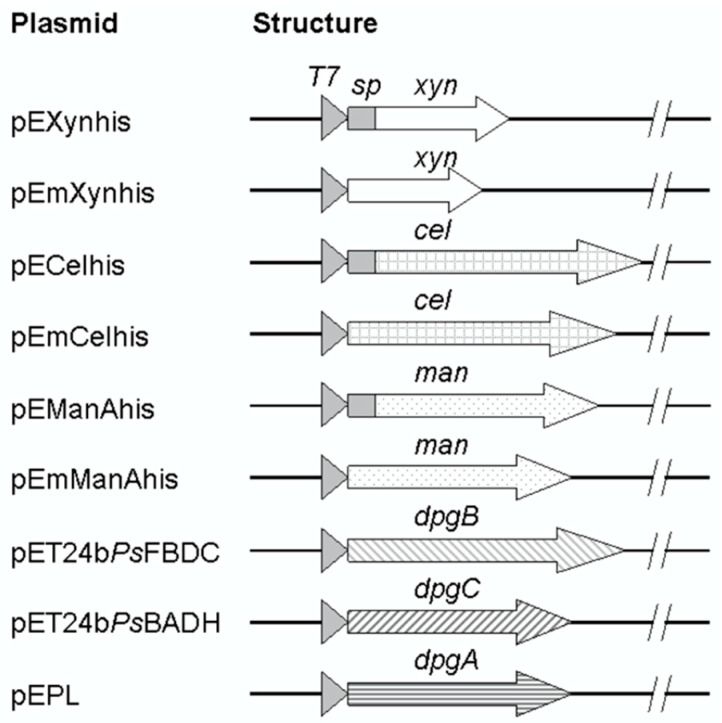
Structures of recombinant plasmids. Secretory enzymes (xylanase, glucanase, and mannanase) corresponding genes were constructed to contain a full-length or only mature part as represented by arrows. DNA regions corresponding to signal peptides were depicted by a gray box located between the *T7* promoter (triangles) and the mature part. β-1,4-xylanase gene; *xyn*, β-1,4-glucanase; *cel*, β-mannanase; *man*, benzoylformate decarboxylase; *dpgB*, benzaldehyde dehydrogenase; *dpgC*, and d-phenylglycine aminotransferase; *dpgA*.

As shown in Figure 1, the full-length genes of *B. subtilis* R5 xylanase gene [GenBank:AB457186] (639 bp) and of *B. subtilis* I15 endo-1,4-glucanase gene [GenBank:FJ464332] (1497 bp) were synthesized and cloned into the *E. coli* expression vector pET24b(+) resulting in pEXynhis, which encoded for the xylanase (with signal peptide, spXyn) containing a C-terminal hexahistidine tag. The plasmid was used as a DNA template for amplification of the mature part of the xylanase corresponding region. The PCR product (555 bp) was then cloned into pET24(+), resulting in pEmXynhis, which encoded for the mature xylanase (Xyn) containing a C-terminal hexahistidine tag. Similarly, the 1497 bp synthetic DNA fragment containing the complete ORF of the glucanase gene was cloned into pET24b(+), resulting in pECelhis, which encoded for the full-length of the glucanase with signal peptide (spCel) with a C-terminal hexahistidine tag. The recombinant plasmid, pECelhis, was further used as a DNA template for amplification and cloning of the mature part of the glucanase corresponding region (1410 bp) by ligation into *Nde*I/*Xho*I site on pET24b(+) resulting in pEmCelhis which encoded for the mature glucanase (Cel) containing a C-terminal hexahistidine tag. Also, pEManAhis containing the full-length gene of a mannanase was used as a DNA template for amplification of the mature part of the mannanase corresponding region (1008 bp). The PCR fragment was cloned into pET24b(+), resulting in pEmManAhis, which encoded for the mature mannanase (Man) containing a C-terminal hexahistidine tag.

All the recombinant plasmids (pEXynhis, pEmXynhis, pECelhis, pEmCelhis, pEManAhis, and pEmManAhis) and those which encoded for intracellular enzymes, including pET24b*Ps*BFDC, pET19b*Ps*BADH [15], and pEPL [20] were verified by restriction enzyme digestion and PCR amplification, and confirmed by nucleotide sequencing.

### 2.2. Expression of Enzymes from Bacillus and Pseudomonas in E. coli

**Figure 2:**
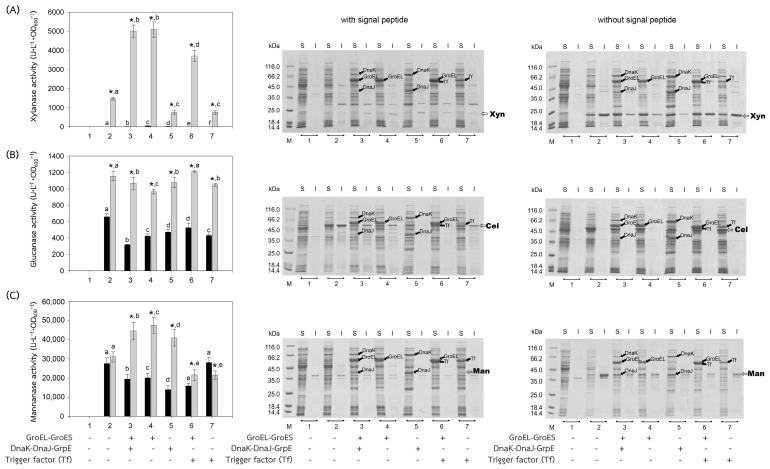
Activities and SDS-PAGE of *Bacillus* enzymes expressed in *E. coli* BL21(DE3). *Bacillus* enzymes, including (**A**) xylanase, (**B**) glucanase, and (**C**) mannanase, were successfully produced in *E. coli* BL21(DE3). Activities of the enzymes expressed with or without their signal peptides were represented by black or gray bars, respectively. (1) Non-induced *E. coli* BL21(DE3) harboring plasmid encoded the enzyme of interest; (2) induced *E. coli* BL21(DE3) harboring plasmid encoded the enzyme of interest, for 3–7, induced *E. coli* BL21(DE3) harboring both plasmids encoded the enzyme of interest and that encoded chaperones (3) GroEL-GroES and DnaK-DnaJ-GrpE, (4) GroEL-GroES, (5) DnaK-DnaJ-GrpE, (6) GroEL-GroES and Trigger factor (Tf), and (7) Trigger factor (Tf). In the case of SDS-PAGE, the enzyme was investigated both in soluble (S) and insoluble fractions (I). Lane M, molecular mass standard marker. Bars represent the standard deviations from triplicate determinations. * represents a significant difference between the activities of the enzyme with and without signal peptide (SP) (*p* < 0.05). Different letters in the same enzyme indicate significant differences (*p* < 0.05).

**Figure 3:**
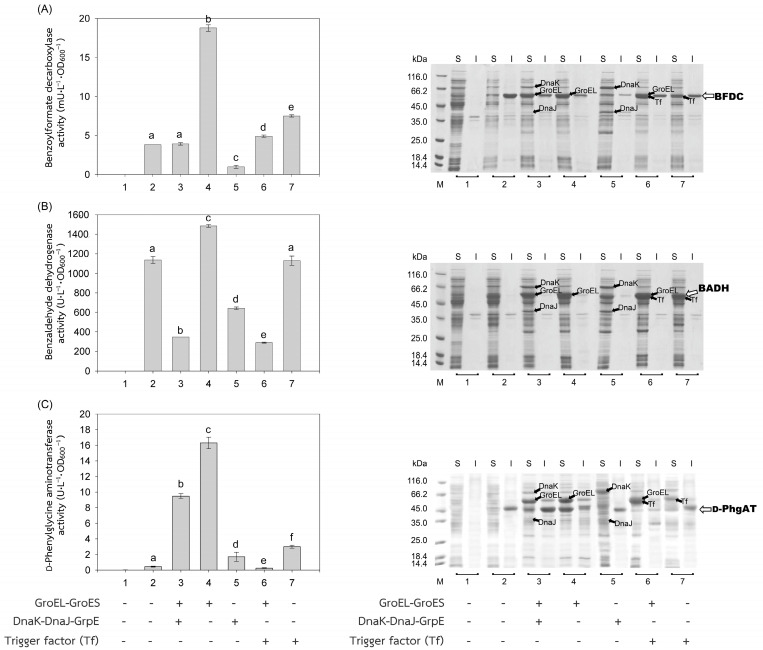
Activities and SDS-PAGE of *Pseudomonas* enzymes expressed in *E. coli* BL21(DE3). Activities of (**A**) benzoylformate decarboxylase (BFDC), (**B**) benzaldehyde dehydrogenase (BADH), and (**C**) d-phenylglycine aminotransferase (d-PhgAT) were depicted by gray bars. Soluble (S) and insoluble proteins (I) were analyzed by SDS-PAGE. (1) Non-induced *E. coli* BL21(DE3) harboring plasmid encoded the enzyme of interest; (2) induced *E. coli* BL21(DE3) harboring plasmid encoded the enzyme of interest, for 3–7, induced *E. coli* BL21(DE3) harboring both plasmids encoded the enzyme of interest and that encoded chaperones (3) GroEL-GroES and DnaK-DnaJ-GrpE, (4) GroEL-GroES, (5) DnaK-DnaJ-GrpE, (6) GroEL-GroES and Trigger factor (Tf), and (7) Trigger factor (Tf). Lane M, molecular mass standard marker. Bars represent the standard deviations from triplicate determinations. Different letters in the same enzyme indicate significant differences (*p* < 0.05).

The recombinant plasmids, pEXynhis, pEmXynhis, pECelhis, pEmCelhis, pEManAhis and pEmManAhis, harboring genes of *Bacillus* secretory enzymes and pET24b*Ps*BFDC, pET19b*Ps*BADH, and pEPL harboring genes of *Pseudomonas* cytoplasmic enzymes were successfully cloned and overexpressed in *E. coli* BL21(DE3) (Table 1). Expression of the secretory enzyme genes whose signal peptide parts were removed was found to be more efficient than those containing the signal peptide parts; that was, yields of Xyn, Cel, and Man were 1468.64 U·L^−1^·OD_600_^−1^, 1154.95 U·L^−1^·OD_600_^−1^ and 30,898.45 U·L^−1^·OD_600_^−1^, which were 1112.61-, 1.75- and 1.12-fold higher than those of spXyn (1.32 U·L^−1^·OD_600_^−1^), spCel, (660.18 U·L^−1^·OD_600_^−1^) and spMan (27,620.03 U·L^−1^·OD_600_^−1^), respectively (Table 1 and Figure 2). The increase in yield of active enzymes might be directly due to the lack of signal peptide, which allows the protein to easily fold into its native form properly either by itself or with the assistance of the cytoplasmic chaperone(s) since the extra signal peptide may interfere both the folding of the protein and the function of the chaperone(s) [21,22]. In addition, the enzyme without signal peptide did not require further processing to be translocated to the cytoplasmic membrane, where its signal peptide was then cleaved to release the mature enzyme into the periplasmic space [23,24]. The results suggested that other extracellular enzymes besides xylanase, glucanase, and mannanase could be successfully folded into their native forms in the cytoplasm of *E. coli* if they were to produce no signal peptide. For intracellular enzymes, over-expression of benzoylformate decarboxylase (BFDC), benzaldehyde dehydrogenase (BADH), and d-phenylglycine aminotransferase (d-PhgAT), which have no signal peptide, were accomplished with the high level of active enzymes 3.80 mU·L^−1^·OD_600_^−1^, 1136.89 U·L^−1^·OD_600_^−1^ and 0.43 U·L^−1^·OD_600_^−1^, respectively (Table 1 and Figure 3).

### 2.3. Effects of Chaperone(s) on Proteins Production in E. coli

In general, co-expression of the GroEL-GroES complex increased the yield of active forms of both intracellular enzymes; BFDC (4.94-fold), BADH (1.31-fold), and d-PhgAT (37.93-fold) (Table 1, Figure 3) and secretory enzymes which contained no signal peptides; xylanase (Xyn) (3.46-fold) and mannanase (Man) (1.53-fold) except that of glucanase where GroEL-GroES complex showed no positive effect (Figure 2). This is as expected since the GroEL-GroES complex function in assisting the correct folding of the protein. Overexpression of the GroEL-GroES complex would increase the helpers; thus, the target proteins will have a higher chance to fold into their native forms. Interestingly, when spMan with its intact signal peptide was co-expressed with the GroEL-GroES complex, the yield of active mannanase decreased from 27,620.03 U·L^−1^·OD_600_^−1^ to 20,113.83 U·L^−1^·OD_600_^−1^. This might be because the chaperones interfered with the normal mannanase maturation process by folding it in a way that decreased and/or delayed the process of translocation to the cell membrane and thereafter. The same negative effect of the GroEL-GroES complex was also seen with glucanase with its intact signal peptide but not that obvious with xylanase (spXyn) with its intact signal peptide since the small values made it less reliable. The exact reason for this is not known, but it was proposed that the nature of Cel, which was highly soluble, might be the reason for finding no inclusion body in the SDS-PAGE and/or the enzyme was relatively easy to renature. Renaturation was determined by heating all the enzyme solutions at 60, 70, and 80 °C for 10 min, then allowing them to renature by incubating them at room temperature for 100 min. The maximal activity of Cel was not diminished even after heating to 60 °C. However, Xyl retained more than 80% of its activity, Man, BFDC, and BADH retained more than 90%, while d-PhgAT lost the majority of its activity. The results are consistent with the prior discovery. After 2 h of incubation, the *B. subtilis* I15 cellulase maintained more than 90% of its maximal activity at 65 °C [25]. On the other hand, the xylanase from *B. subtilis* strain R5 reached 50% inactivation in 25 min at 60 °C and lost nearly all of its activity after 5 min incubation at 75 °C [26]. More than 80% of the mannanase activity from *B. subtilis* BCC41051 was retained after incubation at 60 °C, but it was totally lost following heating at 75 °C for 30 min [27]. The benzoylformate decarboxylase from *P. putida* is stable up to 60 °C for 2 h but rapidly inactivates at 80 °C [16]. The activity of *P. putida* MT53 benzaldehyde dehydrogenase decreased by 50% in 50 min of incubation at 60 °C [28]. d-phenylglycine aminotransferase activity of *P. stutzeri* ST-201 decreased significantly at 60 °C and was entirely inactivated at 70 °C for 10 min [29]. It was found that the activity of Cel recovered higher than the others at all temperatures indicating that Cel had the intrinsic ability to refold correctly better than the others. The other proteins (BFDC, BADH, d-PhgAT, Xyn, and Man), in contrast to Cel, were not only unable to fold into their native forms as easily, but several of them also failed to fold correctly in the absence of chaperones, as demonstrated by the development of inclusion bodies on SDS-PAGE. Hence, overexpression of GroEL-GroES was found to assist in correct protein folding in most cases and therefore increased the yield of active enzymes.

The other chaperones, DnaK-DnaJ-GrpE, increased the production of active Man (1.32-fold) (Figure 2C), which lacks a signal peptide, and d-PhgAT (3.95-fold) (Figure 3C) but decreased the production of Xyn (Figure 2A), BFDC (Figure 3A) and BADH (Figure 3B), whereas no appreciable effects on the production of active Cel (Figure 2B). On the other hand, the Trigger factor (Tf) increased the production of BFDC (1.97-fold) (Figure 3A) and d-PhgAT (6.98-fold) (Figure 3C) but decreased the production of Xyn (Figure 2A) and Man (Figure 2C), and had no effect on the production of active Cel (Figure 2B). Moreover, co-expression of the chaperones GroEL-GroES and DnaK-DnaJ-GrpE (pG-KJE8) or GroEL-GroES and Trigger factor (Tf) (pG-Tf2) did not result in higher yields than those of GroEL-GroES alone, as shown in Table 1. GroEL-GroES decreased in activity when combined with other chaperones compared to when they were produced alone, possibly as a result of an imbalanced number of folding modulators that could also lead to unfavorable proteolytic activities. Previous studies revealed negative consequences for protein productivity and quality when certain chaperones were co-expressed. DnaK was involved in the degradation of aggregation-prone but functional polypeptides by targeting them to proteases such as Lon and ClpP. Due to the proteolysis stimulation caused by DnaK, the yield of recombinant protein was reduced. Additionally, DnaK is a negative regulator of the heat shock response; an increase in DnaK concentration above physiological levels might cause other heat shock proteins to be down-regulated. [30]. On the other hand, the amount of active N-acyl-d-aspartate amidohydrolase (d-AAase) was reduced by the co-expression of GroEL-GroES and Tf [31]. Therefore, the results implied that there were differences in the substrate recognition ability among chaperones as reported data [32,33,34,35,36,37]. Hence, the appropriate selection of chaperones would enhance the yield of active enzymes.

## 3. Materials and Methods

### 3.1. Bacterial Strains, Plasmids, and Culture Conditions

*E. coli* DH5α and *E. coli* BL21(DE3), used for plasmid propagation and over-expression, respectively, were purchased from Novagen (Madison, WI, USA). pET24b(+) was obtained from Novagen (Madison, WI, USA) and was used as a cloning and expression vector. pET24b*Ps*BFDC and pET19b*Ps*BADH containing benzoylformate decarboxylase gene (*dpgB*) and benzaldehyde dehydrogenase gene (*dpgC*) of *Pseudomonas stutzeri* ST201, respectively, were obtained from our previous study [15], and also pEPL containing d-phenylglycine aminotransferase gene (*dpgA*) was obtained from our previous study [20]. Chaperone plasmids pG-KJE8, pGro7, pKJE7, pG-Tf2, and pTf16 were purchased from Takara Bio Inc. (Shiga, Japan). GFX^TM^ PCR DNA and Gel Band Purification Kit were products of GE Healthcare Inc. (Buckinghamshire, UK). Different strains of *E. coli* were cultivated at 37 °C in Luria Bertani (LB) medium (Difco, Tucker, GA, USA).

### 3.2. Synthesis and Cloning of Gene Encoding Full-Length (with Signal Peptide) and Mature Part of Xylanase

An open reading frame (ORF) of a xylanase gene was synthesized by GenScript USA Inc. (Piscataway, NJ, USA) based on the *B. subtilis* R5 xylanase gene [GenBank:AB457186]. The synthesized fragment was designed to contain point mutations from thymine to cytosine at nucleotide position 87 and from adenine to guanine at position 396 in order to eliminate *Nhe*I and *Nde*I recognition sites in the ORF, respectively. In addition, the synthesized fragment was designed to contain oligonucleotide linkers with *Nde*I and *Xho*I recognition sites immediately upstream and downstream of the xylanase gene, respectively. The DNA fragment was ligated into the *Nde*I/*Xho*I linearized pET24b(+), resulting in pEXynhis, which encoded for xylanase (with a signal peptide, spXyn) with a C-terminal hexahistidine tag. Following propagation in *E. coli* DH5α, the recombinant plasmid was then transformed into *E. coli* BL21(DE3) using the standard procedure [38] to allow gene expression.

To construct a xylanase gene containing only the mature protein coding part, primers Xyn-ms-F1 (5′-GTACGCCATATGGCCAGCACAGACTACTGGC-3′, containing a *Nde*I site as underlined) and Xyn-ms-R1 (5′-GTACGCCTCGAGCCACACTGTTACGTTAGAAC-3′, containing an *Xho*I site as underlined) were used to amplify the mature xylanase corresponding region from pEXynhis. The PCR was initially denaturized for 2 min at 95 °C, then underwent 30 cycles of denaturation for 30 s at 95 °C, annealing for 30 s at 50 °C, extension for 1 min at 68 °C, and final extension for 10 min at 68 °C. The PCR fragment was digested with *Nde*I and *Xho*I and then ligated into the corresponding site of the vector pET24(+). The resulting plasmid, pEmXynhis, was expressed for the production of the mature part of xylanase (Xyn) with a C-terminal hexahistidine tag. Each recombinant plasmid was transformed and expressed in *E. coli* BL21(DE3).

### 3.3. Synthesis and Cloning of Gene Encoding Full-Length (with Signal Peptide) and Mature Part of Glucanase

A coding region of the endo-1,4-β-glucanase gene was also synthesized by GenScript USA Inc. (USA) based on the *B. subtilis* I15 endo-1,4-β-glucanase gene [GenBank:FJ464332]. Silent mutation was carried out to change nucleotide residue at position 786 of the ORF from cytosine to thymine, resulting in no *Mlu*I site in the sequence, facilitating future DNA cloning. The synthesized DNA fragment containing the glucanase gene was designed to have *Nde*I and *Xho*I sites upstream and downstream of the ORF, respectively, cloned into the corresponding sites of pET24b(+). The resulting recombinant plasmid, pECelhis, encoded for the glucanase enzyme (with a signal peptide, spCel) with a C-terminal hexahistidine tag.

The mature part of the glucanase corresponding sequence was amplified from pECelhis using oligonucleotides Cel-ms-F1 (5′-GTACGCCATATGGCAGGGACAAAAACGCCAGTAG-3′, containing a *Nde*I site as underlined) and Cel-ms-R1 (5′-GTACGCCTCGAGATTTGGTTCTGTTCCCCAAATC-3′, containing a *Xho*I site as underlined) as primers. The PCR cycling parameters were as follows: initial denaturation at 95 °C for 2 min, followed by 30 cycles of denaturation at 95 °C for 30 s, annealing at 50 °C for 30 s, and extension at 68 °C for 2 min and 50 s before the process is finished with extension at 68 °C for 10 min. After digestion with *Nde*I and *Xho*I, the PCR product was then ligated into the corresponding site of the vector pET24b(+), resulting in pEmCelhis, which encoded for the mature part of the glucanase (Cel) enzyme with a C-terminal hexahidine tag. Each recombinant plasmid was transformed and expressed in *E. coli* BL21(DE3).

### 3.4. Cloning of Gene Encoding Full-Length (with Signal Peptide) and Mature Part of Mannanase

Plasmid pEManAhis containing the full-length of a β-mannanase gene from *Bacillus subtilis* was previously constructed [27] and was used as template DNA for amplification of a region encoding for the mature part of the enzyme. The amplification was performed using primers Man-ms-F1 (5′-GTACGCCATATGCATACTGTGTCGCCTGTGAATCC-3′, containing a *Nde*I site as underlined) and Man-CHR (5′-GTACGCCTCGAGTTCAACGATTGGCGTTAAAGAATC-3′, containing an *Xho*I site as underlined) with the PCR parameter as follow: initial denaturation of the PCR took place at 95 °C for 2 min, followed by 30 cycles of denaturation at 95 °C for 30 s, annealing at 50 °C for 30 s, extension at 68 °C for 2 min, and final extension at 68 °C for 10 min. The PCR product was digested with *Nde*I and *Xho*I and then ligated into the corresponding site of the vector pET-24b(+). The resulting plasmid pEmManAhis was expressed for the mature part of the mannanase (Man) with a C-terminal hexahistidine tag. Each recombinant plasmid was transformed and expressed in *E. coli* BL21(DE3).

### 3.5. Transformation of pET24bPsBFDC, pET19bPsBADH and pEPL

Plasmids pET24b*Ps*BFDC and pET19b*Ps*BADH containing *Pseudomonas stutzeri* ST-201 benzoylformate decarboxylase (*dpgB*) gene, benzaldehyde dehydrogenase (*dpgC*) gene, respectively [15], and pEPL containing d-phenylglycine aminotransferase (*dpgA*) gene were previously constructed [20]. Each of these plasmids was transformed and expressed in *E. coli* BL21(DE3).

### 3.6. Co-Transformation with the Chaperone Plasmid

Plasmids pG-KJE8 (encoded for GroEL-GroES and DnaK-DnaJ-GrpE), pGro7 (encoded for GroEL-GroES), pKJE7 (encoded for DnaK-DnaJ-GrpE), pG-Tf2 (encoded for GroEL-GroES and Trigger factor), and pTf16 (encoded for Trigger factor) [4,39] each was transformed into *E. coli* BL21(DE3) harboring pEXynhis, pEmXynhis, pECelhis, pEmCelhis, pEManAhis, pEmManAhis, pET24b*Ps*BFDC, pET19b*Ps*BADH, and pEPL. Each of the transformants was grown on LB agar plates supplemented with 34 μg·ml^−1^ of chloramphenicol (for selection of the chaperone plasmid) and 30 μg·ml^−1^ of kanamycin (for selection of the expression plasmids pEXynhis, pEmXynhis, pECelhis, pEmCelhis, pEManAhis, pEmManAhis, and pET24b*Ps*BFDC) or 100 μg·ml^−1^ of ampicillin (for selection of the expression plasmid pET19b*Ps*BADH and pEPL). To verify the presence of both recombinant plasmids in the selected *E. coli* transformants, the plasmids were extracted and digested with appropriate restriction enzymes.

### 3.7. Gene Expression

To induce co-expression, a single colony of each transformant was inoculated in 3 mL of LB broth supplemented with appropriate antibiotic(s) and grown overnight at 37 °C with 200 rpm shaking. The overnight pre-cultured was transferred into 50 mL of fresh LB medium in a 250-mL flask with a starting OD_600_ of 0.03. The culture was incubated at 30 °C with 200 rpm shaking until 0.4 OD_600_ was reached. Chaperone(s) expression was induced by adding L-arabinose and/or tetracycline at the final concentration of 0.5 mg·mL^−1^ and/or 5 ng·mL^−1^, respectively, according to the instruction manual provided by Takara Bio Inc. (Shiga, Japan). After 1-h of chaperone(s) induction, synthesis of the target proteins (xylanase, glucanase, mannanase, benzoylformate decarboxylase, benzaldehyde dehydrogenase, and d-phenylglycine aminotransferase) was induced by adding IPTG at a final concentration of 0.4 mM, and the cultures were further incubated (at 30 °C, 200 rpm) for additional 4 h. The cells were collected by 8000 g of centrifugation and washed with 20 mM Tris-HCl buffer (pH 8.0). The cell pellet was resuspended in the same buffer and disrupted by sonication on ice (Vibra-Cell^TM^, Sonics & Materials, Newtown, CT, USA). Cell debris and insoluble aggregates were sedimented at 16,000 g for 30 min at 4 °C. Cell-free extracts containing soluble proteins were used to determine the enzyme activities. Pellets containing insoluble proteins were washed twice with the same buffer. The amount of target proteins, either in soluble or insoluble fractions, was investigated by SDS-PAGE [40].

### 3.8. Protein Determination and SDS-PAGE Analysis

The Bradford method [41] was used to determine the protein content using bovine serum albumin (BSA) as the reference. Protein samples were loaded onto a polyacrylamide gel (12% separating gel and 4% stacking gel). Coomassie Blue staining was applied to the gel following 1.5 h of electrophoresis at 150 V [40].

### 3.9. Enzyme Assays

Xylanase activity; after incubating 0.1 mL of the diluted enzyme sample with 0.9 mL of 0.5% (*w*/*v*) xylan solution in 50 mM sodium phosphate buffer, pH 6.0 at 42 °C for 20 min, the amount of reducing sugars liberated from oat spelt xylan was measured using the dinitrosalicylic acid (DNS) method [26,42]. Absorbance at 540 nm was measured using a spectrophotometer (Unicam Helios Alpha, Thermo, Cambridge, UK). One unit of xylanase activity is defined as the amount of the enzyme required to liberate 1 μmole of reducing sugar equivalent to xylose per min under the assay condition.

Glucanase and mannanase activities were determined using 5% (*w*/*v*) carboxymethyl-cellulose (CMC) (Sigma, Livonia, MI, USA) [25] and 0.5% (*w*/*v*) locust bean gum (LBG) (Sigma, USA) [27] as substrates, respectively, and the DNS method was used to measure the released reducing sugars [42]. One unit of glucanase or mannanase activity was defined as the amount of the enzyme that released 1 μmole of glucose equivalent or mannose equivalent per min under the assay condition, respectively.

Benzoylformate decarboxylase activity was determined using the direct decarboxylase assay by incubating 20 μL of the enzyme sample with 980 μL of the substrate solution containing 100 mM sodium phosphate buffer, pH 6.0, 8.5 mM benzoylformate, 1 mM MgSO_4_, and 40 μM thiamine diphosphate (ThDP). For three minutes at 25 °C, the change in absorbance at 340 nm as benzaldehyde was formed from benzoylformate was continually observed [15,16,43]. One unit of benzoylformate decarboxylase activity is defined as the amount of enzyme that catalyzes the decarboxylation of 1 μmole of benzoylformate per min under the assay condition. The reaction rate was determined with the specific extinction coefficient (ε) of 32 μM^−1^·cm^−1^ for benzoylformate at 340 nm [16].

Benzaldehyde dehydrogenase activity was determined by incubating 20 μL of the enzyme sample with 980 μL of substrate solution containing 100 mM KCl, 100 mM [(2-hydroxy-1,1-bis[hydroxymethyl]ethyl)amino]-1-propanesulfonic acid (TAPS) buffer, pH 8.5, 1 mM benzaldehyde, 1 mM DTT, and 1 mM NAD^+^. For three minutes at 25 °C, the spectrophotometric monitoring of the increase in absorbance at 340 nm as the reduction of NAD^+^ to produce NADH [13,15]. According to the definition of benzaldehyde dehydrogenase activity, one unit is the amount of the enzyme that is necessary to produce 1 μmol of NADH per minute under the assay conditions. For NADH at 340 nm, the reaction rate was calculated using a molar extinction coefficient (ε) of 6220 M^−1^·cm^−1^ [13].

d-phenylglycine aminotransferase activity was determined by measuring the amount of 4-hydroxy benzoylformic acid (4-OHBZF) formed upon transamination of d-4-hydroxyphenylglycine (d-4-OHPhg) using 2-oxoglutarate as an amino acceptor [18,44]. A 20 μL of d-PhgAT solution was incubated with 980 μL of the reaction mixture containing 50 mM 3-(cyclohexylamino)-2-hydroxy-1-propanesulfonic acid buffer, pH 9.5, 10 mM d-4-OHPhg, 10 mM α-ketoglutarate, 5 μM PLP, and 5 μM EDTA. The rate of 4-OHBZF formation was monitored by observing the rise in UV absorption at 340 nm for 3 min at 25 °C. One unit of d-PhgAT activity was defined as the amount of enzyme that catalyzes the formation of 1 μmole of 4-OHBZF per min under the assay condition. The molar absorption coefficient of 4-OHBZF at 340 nm (ε_340nm_) is 2.4 × 10^4^ M^−1^·cm^−1^ [44].

### 3.10. Statistical Analysis

The mean values of enzyme activities were compared using the statistical *t*-test (SPSS statistics 26.0). The *p*-value of 0.05 was used to determine statistical significance.

## 4. Conclusions

A new approach for producing secretory enzymes in *E. coli* was developed. The enzymes tested included xylanase, glucanase, and mannanase, whose yields could be increased when the genes were constructed without their signal peptides corresponding region and expressed in *E. coli* BL21(DE3). Furthermore, when co-expressed with chaperones, especially GroEL-GroES complex, xylanase, and mannanase coding sequences lacking the N-terminal signal peptide regions yielded even higher active enzymes than that with no chaperone co-expression. The benefit of chaperone co-expression was not only with the structural genes of secretory enzymes but also for all the tested intracellular enzymes, BFDE, BADH, and d-PhgAT, whose yields were accomplished in particular, with the GroEL-GroES complex.

## Figures and Tables

**Table 1:** The yield of enzymes expressed in *E. coli* BL21(DE3) and co-expressed with chaperones. Data were expressed as mean ± standard deviation from triplicate determinations. * represents a significant difference between the activities of the enzyme with and without signal peptide (SP) (*p* < 0.05). Different letters in the same row indicate significant differences (*p* < 0.05).

		Enzymes Activities (U·L^−1^·OD_600_^−1^)					
Plasmid	Description	Chaperones Plasmid					
		None	pG-KJE8 (GroEL-GroES, DnaK-DnaJ-GrpE)	pGro7 (GroEL-GroES)	pKJE7 (DnaK-DnaJ-GrpE)	pG-Tf2 (GroEL-GroES, Tf)	pTf16 (Tf)
pEXynhis	Xylanase with SP (spXyn)	1.32 ± 0.04 ^a^	7.74 ± 0.15 ^b^	38.74 ± 1.17 ^c^	15.78 ± 0.39 ^d^	0.54 ± 0.01 ^e^	0.94 ± 0.02 ^f^
pEmXynhis	Xylanase without SP (Xyn)	1468.64 ± 90.66 *^,a^	5002.20 ± 323.16 *^,b^	5083.08 ± 409.19 *^,b^	748.90 ± 87.68 *^,c^	3705.46 ± 372.74 *^,d^	753.91 ± 99.01 *^,c^
pECelhis	Glucanase with SP (spCel)	660.18 ± 38.48 ^a^	318.51 ± 8.11 ^b^	423.05 ± 26.31 ^c^	472.55 ± 33.46 ^d^	525.53 ± 56.60 ^d^	428.72 ± 16.22 ^c^
pEmCelhis	Glucanase without SP (Cel)	1154.95 ± 58.66 *^,a^	1068.83 ± 73.89 *^,b^	964.79 ± 30.40 *^,c^	1077.41 ± 63.79 *^,b^	1211.92 ± 10.12 *^,a^	1048.72 ± 18.19 *^,b^
pEManAhis	Mannanase with SP (spMan)	27,620.03 ± 3029.29 ^a^	19,500.94 ± 2404.23 ^b^	20,113.83 ± 2402.98 ^c^	13,954.43 ± 1866.52 ^d^	15,864.20 ± 1422.00 ^e^	28,100.02 ± 2489.88 ^a^
pEmManAhis	Mannanase without SP (Man)	30,989.45 ± 2704.37 ^a^	44,512.17 ± 4575.60 *^,b^	47,498.04 ± 4225.01 *^,c^	40,933.50 ± 4275.97 *^,d^	21,618.66 ± 2397.71 *^,e^	21,575.18 ± 2139.69 *^,e^
pET24b*Ps*BFDC	Benzoylformate decarboxylase (BFDC) ^#^	3.80 ± 0.01 ^a^	3.91 ± 0.15 ^a^	18.77 ± 0.43 ^b^	0.96 ± 0.15 ^c^	4.91 ± 0.13 ^d^	7.50 ± 0.15 ^e^
pE19b*Ps*BADH	Benzaldehyde dehydrogenase (BADH)	1136.89 ± 36.76 ^a^	346.93 ± 1.06 ^b^	1486.04 ± 13.15 ^c^	641.93 ± 10.52 ^d^	288.11 ± 3.91 ^e^	1129.90 ± 47.30 ^a^
pEPL	d-phenylglycine aminotransferase (d-PhgAT)	0.43 ± 0.01 ^a^	9.49 ± 0.31 ^b^	16.31 ± 0.74 ^c^	1.70 ± 0.52 ^d^	0.25 ± 0.03 ^e^	3.00 ± 0.17 ^f^

^#^ The activity of benzoylformate decarboxylase (BFDC) is shown in mU·L^−1^·OD600^−1^.

## Data Availability

Not applicable.

## Sample Availability

Samples of the compounds are available from the authors.
